# The scaling and allometry of organ size associated with miniaturization in insects: A case study for Coleoptera and Hymenoptera

**DOI:** 10.1038/srep43095

**Published:** 2017-02-22

**Authors:** Alexey A. Polilov, Anastasia A. Makarova

**Affiliations:** 1 Department of Entomology, Faculty of Biology, Lomonosov Moscow State University, Moscow 119234, Russia

## Abstract

The study of the influence of body size on structure in animals, as well as scaling of organs, is one of the key areas of functional and evolutionary morphology of organisms. Most studies in this area treated mammals or birds; comparatively few studies are available on other groups of animals. Insects, because of the huge range of their body sizes and because of their colossal diversity, should be included in the discussion of the problem of scaling and allometry in animals, but to date they remain insufficiently studied. In this study, а total of 28 complete (for all organs) and 24 partial 3D computer reconstructions of body and organs have been made for 23 insect species of 11 families and five orders. The relative volume of organs was analyzed based on these models. Most insect organs display a huge potential for scaling and for retaining their organization and constant relative volume. By contrast, the relative volume of the reproductive and nervous systems increases by a considerable factor as body size decreases. These systems can geometrically restrain miniaturization in insects and determine the limits to the smallest possible body size.

Body size is a parameter of utmost importance; it largely determines the morphology, physiology, and biology of organisms^1^. Changes in body size, especially extreme miniaturization, can result in considerable reorganizations of structure and changes of proportions of the body and organs^2^. The problem of allometry and scaling of organs is the subject of many studies, but most of them treat only mammals and birds (for review, see^3^). Insects are very convenient for studying the scaling of organs and tissues which is associated with extreme miniaturization: first, they have a huge range of body sizes (the largest is more than 2000 times as long as the smallest); second, the smallest insects are comparable in size to unicellular organisms but retain high morphological complexity^4^. However, scaling of organs in insects remains studied insufficiently; only studies on particular organs are available, above all on brain size^5^ and only some data on the relative volume of all organs in several species^6^,^7^. The purpose of this study was to provide the large-scale analysis of scaling in insect organs and miniaturization-related allometry of organs. The focus will be on the Coleoptera and Hymenoptera, which include a significant part of microinsects and the smallest known insects^4^.

## Results and Discussion

A total of 28 complete (for all organs) and 24 partial 3D computer reconstructions of body and organs have been made for 23 insect species of 11 families and five orders (Thysanura, Psocoptera, Thysanoptera, Coleoptera, and Hymenoptera) including the smallest insects *Megaphragma* and *Nanosella* and large representatives of related taxa (the body length and body volume differed by a factor of up to 100 and about 100 000, respectively) (Table S1). The volume of organs was analyzed based on these models.

### The volume of the skeleton

**Figure 1:**
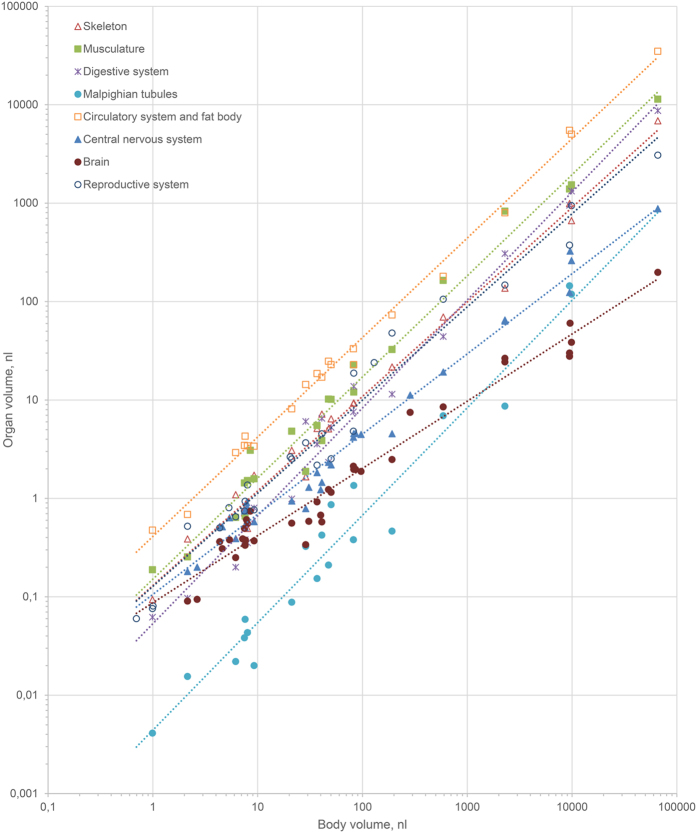
Scaling of organ size in adult insects.

**Figure 2:**
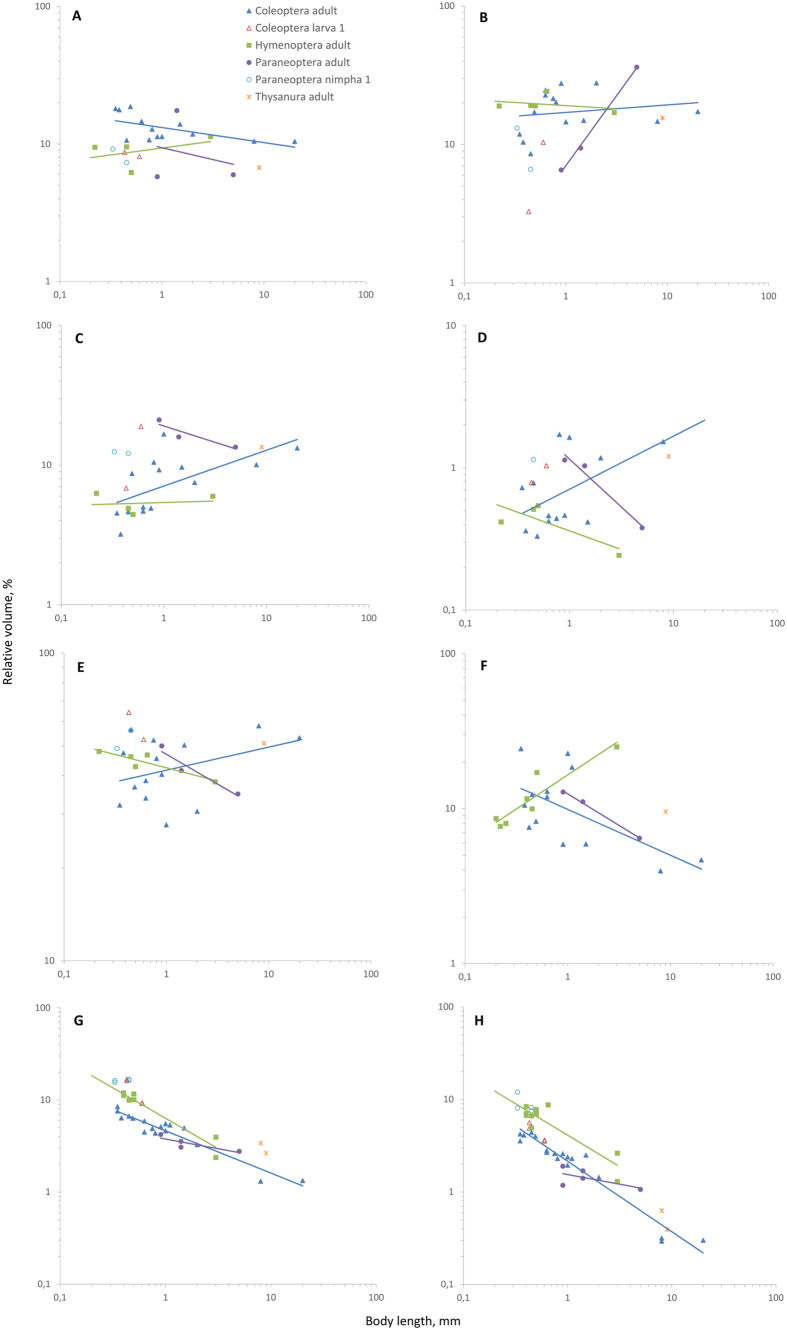
Changes in relative volume of organs in adults and larvae of insects of different taxa. (**A**) skeleton, (**B**) musculature, (**C**) digestive system, (**D**) Malpighian tubules, (**E**) circulatory system and fat body, (**F**) reproductive system, (**G**) central nervous system, (**H**) brain.

In adult insects generally changes proportionally to the body volume, i.e., isometrically (slope of regression is not significantly different from 1, Fig. 1, Table 1). Analysis of changes in this parameter in different groups shows (Fig. 2A) that the relative volume of the skeleton slightly increases in Coleoptera and Paraneoptera and slightly decreases in Hymenoptera as body size decreases. In spiders the weight of the skeleton markedly decreases with decreasing body size^8^. Interestingly, the slope of scaling of the skeleton in insects is much closer to 1 than in mammals (1.090^9^ to 1.142^10^) or birds (1.071^9^), which gives evidence that the insect skeleton is scaled more proportionally.

### The volume of the musculature

In insects generally changes isometrically (Fig. 1, Table 1). Decreasing relative volume with decreasing body size is found in Coleoptera and Paraneoptera (Fig. 2B). Since relative muscle strength is determined by cross-section area, which changes with linear body size at a lower rate than volume, relative muscle strength increases as body size decreases^11^, making it possible to increase the relative volume of the musculature. Hymenoptera are exceptional: the relative volume of their musculature slightly increases as body size decreases (Fig. 2B). Increased relative area of flight musculature in cross-sections has been shown for small dipterans^12^, but since no data on volumes are available for these insects, it is difficult to make conclusions about allometry. In mammals the musculature also changes isometrically (slope 0.99–1.00), and in birds its relative weight decreases as body size decreases (slope 1.08)^3^.

### The volume of the digestive system

In insects generally changes allometrically (slope of regression is significantly different from 1, Fig. 1, Table 1). The relative volume decreases in Coleoptera, almost unchanged in Hymenoptera, and increases in Paraneoptera as body size decreases (Fig. 2C). Isometry and decrease can be explained by the gut efficiency, determined by surface area, which changes with body size at a lower rate than volume, so that efficiency increases as body size decreases^13^. The slope of the digestive system, including the gut content, is similar in insects to that of mammals (1.062) and much smaller than in birds (1.204) or other reptiles (1.389)^14^. Malpighian tubules generally show similar trends as the digestive system, except in Hymenoptera, in which their relative volume increases as body size decreases (Figs 1 and 2D).

### The volume of the circulatory system and fat body

In insects generally changes isometrically (Fig. 1, Table 1). Analysis of separate taxa shows that the relative volume of this system somewhat increases in Hymenoptera and Paraneoptera, and decreases in Coleoptera as body size decreases (Fig. 2E). The decrease found in Coleoptera probably reflects the almost complete absence of the circulatory system of Ptiliidae^4^. Since the volume of the circulatory system in insects depends on many factors (amount of reserve substances, degree of reproductive products development, age, etc.^15^), data on the types of changes within orders can change as new, more extensive material becomes available.

Because of the extremely small diameter of the tracheae, the volume of the respiratory system could not be calculated, but considering the strong reduction of this system in microinsects^4^, it can be assumed that its relative volume decreases as body size decreases, which is compensated by the higher passive respiration efficiency in smaller insects.

### The volume of the central nervous system

Strongly changes allometrically as body size decreases (Fig. 1, Table 1); in insects generally and all studied taxa, the relative volume of this system strongly increases as body size decreases (Fig. 2G). The greatest relative volume of this system among adult insects is found in Hymenoptera (about 12%). First-instar larvae of Coleoptera and first-instar nymphs of Paraneoptera have considerably greater relative volumes of this system compared to adults. The relative volume of this system in the first-instar nymph of the thrips *Heliothrips haemorrhoidalis* is about 17%, which is almost 5 times as great as in the adult. Increasing relative size of this system has also been shown in other small arthropods^5^,^12^,^16^,^17^,^18^,^19^.

The relative volume of the brain (cerebral index), widely used in discussions of the evolution of neural activity in animals^1^, should be considered separately. The relative weight of the human brain is 2.5%; it was long believed that cerebral index among animals was the highest in hummingbirds, 8.33%, but it reaches 8.36% in the miniature hymenopteran *Trichogramma*, 11.95% in first-instar nymphs of the psocopteran *Liposcelis,* and 15% in *Brachymyrmex,* some of the smallest ants^20^. The rule of brain size changing allometrically with body size, known as Haller’s rule or brain–body allometry, has been confirmed for many vertebrates^21^,^22^,^23^,^24^,^25^,^26^, insects^5^,^12^,^20^,^27^,^28^,^29^,^30^, spiders^19^, or other invertebrates^5^; it is fully realized in the smallest insects; moreover, our results considerably broaden the scope of this rule. The slope of the brain in insects is comparable to that of mammals, in which it varies, according to different data, from 0.664 to 0.76^3^. The only exceptions to Haller’s rule are several laboratory lines of *Trichogramma*^30^. *Megaphragma* also has markedly smaller relative brain volume than larger representatives of related taxa, because of the anucleate neurons, a unique feature of this genus^31^; therefore, data on the volume of the cerebrum and of the central nervous system of *Megaphragma* were excluded from the main analysis. Data on the link between the complexity of behavior and brain size in vertebrates are extremely interesting, but among insects such data are available only for social species^5^,^32^,^33^. The behavior of microinsects never became subject of special studies, but all principal behaviors typical of large representatives of related groups are found also in microinsects. It has also been shown that in microscopic spiders body size diminution does not result in simplification of behavior^34^,^35^.

### The reproductive system

In insects generally changes allometrically and increases in relative volume as body size decreases (Fig. 1, Table 1). The same trend is found in Coleoptera and Paraneoptera (Fig. 2F). Increasing relative volume of the reproductive system in free-living insects is associated with the relative egg size strongly increasing as body size decreases^4^. The relative volume of the reproductive system varies considerably among coleopterans, apparently because of the dependence of this parameter on the reproductive stage. Negative allometry has also been shown in linear measurements of the genital apparatus of many insects and spiders^36^. The gonads have a much greater slope in insects than in birds, in which it is 0.76–0.92^3^. Among hymenopterans, this parameter markedly decreases as body size decreases, because in egg parasitoids the relative size of the egg shows no distinct increase: their larvae, which develop in the host egg, are strongly de-embryonized and consume little or no yolk^37^.

Thus, most insect organs show huge scaling potentials, retaining their organization^4^ and even relative volume as body size decreases to small fractions of its initial values. The skeleton, musculature, and circulatory systems change isometrically over the entire studied series of insects. The relative volume of those organs the efficiency of which is determined by area (the digestive system and Malpighian tubules) or diffusion rate (the tracheal system), parameters that increase as body size decreases, is smaller in smaller insects, but the relative volumes of the reproductive and nervous systems strongly increases as body size decreases. These systems can geometrically restrain miniaturization in insects and determine the limits of the smallest possible body size. The greatest rate of relative volume increase at decreasing body size is found in the nervous system; in the smallest insects this parameter reaches nearly one-fifth, making the cerebral index (relative brain weight) considerably greater than in any animals for which it is known, including humans. Although the range of body sizes in the studied insects is comparable to that of vertebrates, most organ systems in insects demonstrate much smaller allometry, suggesting that the organization of insects has a greater scaling capacity.

## Methods

### Experimental procedures

The material was fixed in FAE (formaldehyde, acetic acid, ethanol) and embedded in Araldite by the standard method. The resulting blocks were used to make complete series of cross-sections and longitudinal sections 1 μm for specimens less than 2 mm long and 2–4 μm for specimens more than 2 mm long. For 3D computer modeling, the series of sections were photographed under a Motic BA410 microscope. After alignment of sections and calibration of the resulting stack, the body and organs were modeled in the program Bitplane Imaris. All structures were outlined manually and automatically recalculated as three-dimensional. Most of the 3D reconstructions used in this study have been published in earlier studies on the morphology of various microinsects (review^4^, animated 3D-pdf^38^ and etc.). The volume of organs and body (without legs, wings, antennae, and other appendages) was calculated using 3D reconstructions in Bitplane Imaris statistical module. The volume of the skeleton (legs, wings, antennae, and other appendages) was calculated based on the area of the integument and average thickness of the cuticle calculated from 80 measurements (eight in each of ten equidistant sections evenly distributed over the body). The volume of the fat body and hemolymph was determined as the difference between the body volume and total volumes of all organs. The obtained volumes were analyzed in R with a SMATR 3 package using the Standardized Major Axis (SMA) and Ordinary Least Squares (OLS) estimation (which are usually used for analyzing morphological allometry^39^). Non-independence of the data was controlled in the ape package^40^ using Phylogenetic Generalized Least Squares (PGLS) for samples of one specimen per species and in the MCMCglmm package^41^ using Markov chain Monte Carlo generalized linear mixed models (MCMCglmm) for samples of more than one specimen per species. The phylogenetic data were obtained from the Open Tree of Life online database^42^.

### List of taxa examined

**Thysanura Latreille, 1796**

**Lepismatidae Latreille, 1802**

*Lepisma saccharina* Linnaeus, 1758

**Psocoptera Shipley, 1904**

**Liposcelididae Enderlein, 1911**

*Liposcelis bostrychophila Badonnel 1931*

**Psocidae Shipley, 1904**

*Copostigma sp.*

**Thysanoptera Haliday, 1836**

**Thripidae Stevens, 1829**

*Heliothrips haemorrhoidalis* (Bouché, 1833)

**Coleoptera Linnaeus, 1758**

**Ptiliidae Erichson, 1845**

*Nanosella russica* Polilov, 2008

*Nanosella* sp.

*Primorskiella anodonta* Polilov, 2008

*Porophila mystacea* Polilov, 2008

*Mikado* sp.

*Acrotrichis grandicollis* (Mannerheim, 1844).

*Acrotrichis montandoni* (Allibert, 1844)

**Hydraenidae Mulsant, 1844**

*Ochthebius sp.*

**Staphylinidae Lameere, 1900**

*Atheta* sp.

*Aleochara* sp.

*Staphylinus caesareus* Cederhjelm, 1798.

**Corylophidae LeConte, 1852**

*Sericoderus lateralis* (Gyllenhal, 1827)

*Orthoperus atomus* (Gyllenhal, 1808)

**Hymenoptera Linnaeus, 1758**

**Mymaridae Haliday, 1833**

*Anaphes flavipes* (Förster, 1841)

*Anagrus* sp.

**Trichogrammatidae Haliday et Walker, 1851**

*Trichogramma evanescens* Westwood, 1833

*Trichogramma* sp.

*Megaphragma mymaripenne* Timberlake, 1924

**Eulophidae Westwood, 1829**

*Hemiptarsenus* sp.

## Additional Information

**How to cite this article**: Polilov, A. A. and Makarova, A. A. The scaling and allometry of organ size associated with miniaturization in insects: A case study for Coleoptera and Hymenoptera. *Sci. Rep.* **7**, 43095; doi: 10.1038/srep43095 (2017).

**Publisher's note:** Springer Nature remains neutral with regard to jurisdictional claims in published maps and institutional affiliations.

## Supplementary Material

Supplementary Information

## Figures and Tables

**Table 1:** Scaling of organ volume as a function body volume in insects.

Taxon	Slope			
	OLS	SMA	PGLS	MCMCglmm
Skeleton				
Insects, all	0.975	0.979	0,987	0.968
Coleoptera	0.967*	0.965*	0,988	0.965
Hymenoptera	1.055	1.050	1,044	—
Paraneoptera	0.923	0.893	0,888	—
Musculature				
Insects, all	1.038	1.029	1,057	1.050
Coleoptera	1.041	1.033	1,059	1.045
Hymenoptera	0.974	0.972	0,966	—
Paraneoptera	1.367*	1.367*	1,366*	—
Digestive system				
Insects, all	1.109***	1.099**	1,059	1.063*
Coleoptera	1.108**	1.101**	1,065	1.076
Hymenoptera	1.010	1.007	1,012	**—**
Paraneoptera	0.923	0.922	0,923	—
Malpighian tubules				
Insects, all	1.111**	1.089*	1,028	1.063
Coleoptera	1.183**	1.160**	1,055	1.131
Hymenoptera	0.892	0.884	0,871	—
Paraneoptera	0.749***	0.749***	0,749***	—
Circulatory system and fat body				
Insects, all	1.013	1.010	1,014	1.011
Coleoptera	1.030	1.026	1,024	1.026
Hymenoptera	0.955**	0.955**	0,951**	—
Paraneoptera	0.934	0.933	0,934	—
Central nervous system				
Insects, all	0.833***	0.821***	0,813***	0.821***
Coleoptera	0.838***	0.830***	0,82**	0.825***
Hymenoptera	0.665***	0.657***	0,564**	0.685**
Paraneoptera	0.960	0.958	0,974	1.058
Brain				
Insects, all	0.697***	0.683***	0,706***	0.714***
Coleoptera	0.699***	0.694***	0,716***	0.707***
Hymenoptera	0.662***	0.640***	0,480**	0.656**
Paraneoptera	0.929	0.926	0,956	0.997
Reproductive system				
Insects, all	0.961*	0.948*	0,878*	0.918*
Coleoptera	0.907*	0.891**	0,838*	0.876*
Hymenoptera	1.212**	1.209***	1,235**	1.205**
Paraneoptera	0.852*	0.852*	0,852*	—

For complete version of this table, which includes elevation, R^2^, and CI 95%, and for table on the relative volumes of organs, see Supplementary material (Tables S2 and S3).*p-value for slope different from 1, 0.05 ≤ p < 0.1; **0.01 ≤ p < 0.05; ***p < 0.01.
