# Supplementary material for: The scaling and allometry of organ size associated with miniaturization in insects: A case study for Coleoptera and Hymenoptera
Source: Sci Rep. 2017 Feb 22;7:43095. doi: 10.1038/srep43095 (PMC5320524; doi:10.1038/srep43095)
Supplement: Supplementary Information [file srep43095-s1.pdf]

The scaling and allometry of organ size associated with miniaturization in insects: A case study for Coleoptera and Hymenoptera

Alexey A. Polilov*, Anastasia A. Makarova

Department of Entomology, Faculty of Biology, Lomonosov Moscow State University, Moscow 119234, Russia

* Correspondence to polilov@gmail.com

Table S1. Volumes of organs

Taxon	Stage	Body length, mm	Body volume, nl	Organ volume, nl							
				Skeleton	Musculature	Digestive system	Malpighian tubules	Circulatory system and fat body	Central nervous system	Brain	Reproductive system
Lepisma saccharina	Adult	9.0	9867.5	663.9	1534.0	1326.6	118.7	5021.9	260.5	38.5	941.9
Lepisma saccharina	Adult	8.0	9579.8	–	–	–	–	–	325.9	60.3	–
Liposcelis bostrychophila	Adult	0.9	28.714	1.658	1.881	6.049	0.325	14.341	0.787	0.338	3.673
Liposcelis bostrychophila	Adult	0.9	30.802	–	–	–	–	–	1.296	0.584	–
Liposcelis bostrychophila	Nymph 1 ins	0.33	2.284	0.210	0.300	0.285	0.000	1.119	0.370	0.184	–
Liposcelis bostrychophila	Nymph 1 ins	0.33	2.525	–	–	–	–	–	0.392	0.302	–
Copostigma sp.	Adult	5.0	2289.7	136.7	828.3	307.4	8.7	798.1	63.4	24.3	147.2
Copostigma sp.	Adult	5.0	2286.4	–	–	–	–	–	65.0	26.5	–
Heliothrips haemorrhoidalis	Adult	1.4	40.99	7.18	3.86	6.52	0.42	17.02	1.45	0.58	4.53
Heliothrips haemorrhoidalis	Adult	1.4	39.959	–	–	–	–	–	1.225	0.675	–
Heliothrips haemorrhoidalis	Nymph 1 ins	0.45	3.176	0.232	0.211	0.385	0.036	1.781	0.531	0.238	–
Heliothrips haemorrhoidalis	Nymph 1 ins	0.45	3.090	–	–	–	–	–	0.518	0.259	–
Atheta sp.	Adult	1.5	36.880	5.131	5.503	3.560	0.153	18.531	1.827	0.921	2.175
Ochthebius sp.	Adult	2.0	589.5	69.5	163.9	44.2	6.9	180.4	19.2	8.5	105.4
Aleochara sp.	Adult	8.0	9471.9	990.8	1391.6	952.0	144.6	5494.1	123.9	27.8	374.9
Staphylinus caesareus	Adult	8.0	9471.9	–	–	–	–	–	–	30.0	–
Staphylinus caesareus	Adult	20	65850.3	6865.5	11378.0	8710.0	–	34949.8	876.9	197.9	3070.1
Acrotrichis grandicollis	Adult	0.9	82.03	9.26	22.69	7.58	0.38	33.11	4.19	2.12	4.82
Acrotrichis grandicollis	Adult	1.0	96.94	–	–	–	–	–	4.460	1.880	–
Acrotrichis montandoni	Adult	0.75	47.47	5.08	10.20	2.33	0.21	24.75	2.32	1.23	–
Mikado sp.	Adult	0.45	7.600	0.800	0.660	0.380	0.059	4.284	0.506	0.334	0.934
Mikado sp.	Adult	0.43	6.320	–	–	–	–	–	–	–	0.804
Mikado sp.	Adult	0.45	7.200	–	–	–	–	–	–	0.388	–
Mikado sp.	Larva 1 ins	0.43	0.432	0.038	0.014	0.030	0.003	0.277	0.070	0.024	–
Mikado sp.	Larva 1 ins	0.43	0.441	–	–	–	–	–	0.074	0.022	–
Mikado sp.	Larva 3 ins	0.84	0.863	0.118	0.029	0.083	0.010	0.582	0.040	0.018	–
Nanosella russica	Adult	0.35	2.141	0.387	0.254	0.097	0.015	0.686	0.181	0.090	0.520
Nanosella russica	Adult	0.35	2.648	–	–	–	–	–	0.200	0.094	–
Nanosella sp.	Adult	0.38	6.170	1.090	0.640	0.200	0.022	2.920	0.390	0.250	0.650
Primorskiella anodonta	Adult	0.49	9.240	1.720	1.580	0.800	0.031	3.390	0.580	0.370	0.765
Porophilla mystacea	Adult	0.63	21.130	3.080	4.810	0.990	0.088	8.130	0.940	0.560	2.515
Porophilla mystacea	Adult	0.62	20.450	2.965	5.045	1.023	0.094	6.908	1.207	0.573	2.636
Sericoderus lateralis	Adult	1.0	82.60	9.34	12.03	13.76	1.35	22.84	4.56	1.97	18.72
Sericoderus lateralis	Adult	1.1	86.047	–	–	–	–	–	4.582	1.969	–
Sericoderus lateralis	Adult	1.1	129.406	–	–	–	–	–	–	–	23.924
Sericoderus lateralis	Larva 4 ins	1.7	68.317	8.537	6.886	12.235	1.030	38.639	0.991	0.343	–
Sericoderus lateralis	Larva 1 ins	0.6	5.536	0.449	0.575	1.045	0.057	2.903	0.507	0.196	–
Sericoderus lateralis	Larva 1 ins	0.6	5.495	–	–	–	–	–	0.509	0.199	–
Orthoperus atomus	Adult	0.8	50.284	6.439	10.149	5.261	0.863	22.860	2.189	1.152	2.524
Anaphes flavipes	Adult	0.45	7.486	0.713	1.433	0.366	0.038	3.446	0.744	0.496	0.745
Anaphes flavipes	Adult	0.45	7.658	–	–	–	–	–	0.763	0.376	–
Anaphes flavipes	Adult	0.4	5.383	–	–	–	–	–	0.635	0.379	–
Trichogramma sp.	Adult	0.5	7.994	0.495	1.518	0.354	0.043	3.416	0.803	0.554	1.365
Trichogramma sp.	Adult	0.5	7.830	–	–	–	–	–	0.909	0.607	–
Trichogramma evanescens	Adult	0.4	4.343	–	–	–	–	–	0.518	0.363	0.502
Trichogramma evanescens	Adult	0.4	4.602	–	–	–	–	–	0.515	0.309	–
Anagrus sp.	Adult	0.65	8.512	–	2.079	–	–	–	–	0.742	–
Hemiptarsenus sp.	Adult	3.0	191.61	21.65	32.56	11.45	0.46	73.04	4.54	2.48	47.91
Hemiptarsenus sp.	Adult	3.0	285.06	–	–	–	–	–	11.21	7.47	–
Megaphragma mymaripenne	Adult	0.25	0.992	0.094	0.188	0.062	0.004	0.475	0.082	0.045	0.076
Megaphragma mymaripenne	Adult	0.22	0.804	–	–	–	–	–	0.054	0.037	0.081
Megaphragma mymaripenne	Adult	0.22	0.696	–	–	–	–	–	0.052	0.029	0.060

Table S2. Scaling of organ volume and body volume in adult insects.

Taxon	SMA				OLS				PGLS				MCMCglmm			
	Elevation	Slope	R^2	CI for slope 95%	Elevation	Slope	R^2	CI for slope 95%	Intercept	Slope	R^2	CI for slope 95%	Elevation	Slope	R^2	CI for slope 95%
**Skeleton**																
Ins.	–0.925	0.975	0.987	0.924<br>1.028	–0.913	0.979	0.987	0.922<br>1.037	–1.009	0.987	0.983	0.927<br>1.047	–0.959	0.968	0.985	0.924<br>1.017
Col.	–0.836	0.967 *	0.996	0.931<br>1.004	–0.839	0.965 *	0.998	0.929<br>1.006	–0.889	0.988	0.982	0.910<br>1.066	–0.837	0.965	0.995	0.920<br>1.015
Hym.	–1.110	1.055	0.990	0.789<br>1.412	–1.105	1.050	0.990	0.739<br>1.362	–1.051	1.044	0.999	1.028<br>1.060	–	–	–	–<br>–
Par.	–0.909	0.923	0.934	0.139<br>6.144	–0.844	0.893	0.934	–2.109<br>3.896	–0.796	0.888	0.952	0.500<br>1.276	–	–	–	–<br>–
**Musculature**																
Ins.	–0.840	1.038	0.981	0.976<br>1.105	–0.823	1.029	0.981	0.965<br>1.093	–0.929	1.057	0.973	0.979<br>1.135	–0.912	1.050	0.980	0.981<br>1.124
Col.	–0.842	1.041	0.986	0.967<br>1.120	–0.829	1.033	0.986	0.957<br>1.110	–0.900	1.059	0.973	0.956<br>1.162	–0.855	1.045	0.985	0.958<br>1.137
Hym.	–0.682	0.974	0.995	0.862<br>1.101	–0.680	0.972	0.995	0.852<br>1.091	–0.681	0.966	0.997	0.893<br>1.039	–	–	–	–<br>–
Par.	–1.672	1.367 *	0.998	0.888<br>2.106	–1.670	1.367 *	0.998	0.757<br>1.976	–1.661	1.366 *	0.999	1.287<br>1.445	–	–	–	–<br>–
**Digestive system**																
Ins.	–1.304	1.109 ***	0.980	1.040<br>1.183	–1.284	1.099 **	0.980	1.027<br>1.170	–1.108	1.059	0.971	0.976<br>1.142	–1.125	1.063 *	0.979	0.987<br>1.133
Col.	–1.339	1.108 **	0.985	1.029<br>1.194	–1.325	1.101 **	0.985	1.018<br>1.183	–1.188	1.065	0.969	0.954<br>1.176	–1.213	1.076	0.984	0.978<br>1.164
Hym.	–1.286	1.010	0.994	0.815<br>1.251	–1.284	1.007	0.994	0.789<br>1.225	–1.264	1.012	0.998	0.933<br>1.091	–	–	–	–<br>–
Par.	–0.618	0.923	0.996	0.473<br>1.802	–0.615	0.922	0.996	0.258<br>1.586	–0.626	0.923	0.997	0.837<br>1.009	–	–	–	–<br>–
**Malpighian tubules**																
Ins.	–2.386	1.111 **	0.960	1.010<br>1.222	–2.348	1.089 *	0.960	0.983<br>1.195	–2.215	1.028	0.931	0.895<br>1.161	–2.256	1.063	0.956	0.960<br>1.178
Col.	–2.474	1.183 **	0.960	1.038<br>1.349	–2.436	1.160 **	0.960	1.004<br>1.316	–2.163	1.055	0.928	0.873<br>1.237	–2.298	1.131	0.954	0.967<br>1.287
Hym.	–2.284	0.892	0.981	0.598<br>1.331	–2.276	0.884	0.981	0.517<br>1.251	–2.294	0.871	0.990	0.703<br>1.039	–	–	–	–<br>–
Par.	–1.582	0.749 ***	0.999	0.740<br>0.758	–1.582	0.749 ***	0.999	0.740<br>0.758	–1.582	0.749 ***	1	0.748<br>0.750	–	–	–	–<br>–
**Circulatory system and fat body**																
Ins.	–0.393	1.013	0.995	0.981<br>1.046	–0.389	1.010	0.995	0.978<br>1.043	–0.397	1.014	0.989	0.966<br>1.062	–0.386	1.011	0.994	0.975<br>1.048
Col.	–0.433	1.030	0.993	0.980<br>1.082	–0.427	1.026	0.993	0.976<br>1.077	–0.430	1.024	0.988	0.955<br>1.093	–0.444	1.026	0.993	0.961<br>1.090
Hym.	–0.310	0.955 **	0.999	0.919<br>0.992	–0.310	0.955 **	0.999	0.918<br>0.991	–0.304	0.951 **	0.999	0.934<br>0.968	–	–	–	–<br>–
Par.	–0.239	0.934	0.998	0.602<br>1.448	–0.238	0.933	0.998	0.511<br>1.356	–0.245	0.934	0.999	0.880<br>0.988	–	–	–	–<br>–
**Central nervous system**																
Ins.	–1.020	0.833 ***	0.972	0.783<br>0.885	–0.999	0.821 ***	0.972	0.770<br>0.872	–1.000	0.813 ***	0.912	0.696<br>0.930	–0.978	0.821 ***	0.972	0.765<br>0.879
Col.	–1.075	0.838 ***	0.982	0.777<br>0.903	–1.061	0.830 ***	0.982	0.767<br>0.894	–1.038	0.82 **	0.907	0.665<br>0.975	–1.044	0.825 ***	0.999	0.748<br>0.893
Hym.	–0.702	0.665 ***	0.974	0.577<br>0.768	–0.692	0.657 ***	0.974	0.561<br>0.752	–0.631	0.564 **	0.999	0.543<br>0.585	–0.728	0.685 **	0.970	0.450<br>0.926
Par.	–1.416	0.960	0.995	0.872<br>1.057	–1.411	0.958	0.995	0.865<br>1.050	–1.459	0.974	0.997	0.884<br>1.064	–2.068	1.058	0.993	0.228<br>4.444
**Brain**																
Ins.	–1.089	0.697 ***	0.960	0.651<br>0.746	–1.063	0.683 ***	0.960	0.635<br>0.731	–1.176	0.706 ***	0.966	0.647<br>0.765	–1.146	0.714 ***	0.960	0.658<br>0.772
Col.	–1.137	0.699 ***	0.985	0.658<br>0.744	–1.128	0.694 ***	0.985	0.651<br>0.737	–1.158	0.716 ***	0.976	0.650<br>0.782	–1.131	0.707 ***	0.986	0.645<br>0.762
Hym.	–0.889	0.662 ***	0.933	0.537<br>0.816	–0.864	0.640 ***	0.933	0.501<br>0.779	–0.696	0.480 **	0.975	0.366<br>0.594	–0.876	0.656 **	0.925	0.434<br>0.922
Par.	–1.713	0.929	0.993	0.828<br>1.042	–1.706	0.926	0.993	0.819<br>1.033	–1.818	0.956	0.998	0.891<br>1.021	–1.859	0.997	0.991	0.504<br>1.544
**Reproductive system**																
Ins.	–0.907	0.961*	0.973	0.896<br>1.002	–0.887	0.948 *	0.973	0.881<br>1.015	–0.679	0.878 **	0.930	0.764<br>0.992	–0.772	0.918 *	0.969	0.820<br>1.011
Col.	–0.803	0.907 *	0.966	0.809<br>1.016	–0.775	0.891 **	0.966	0.788<br>0.995	–0.624	0.838 *	0.929	0.687<br>0.989	–0.676	0.876 *	0.958	0.738<br>1.007
Hym.	–1.077	1.212 **	0.995	1.120<br>1.312	–1.076	1.209 **	0.995	1.113<br>1.305	–1.149	1.235 **	0.998	1.171<br>1.299	–1.088	1.205 **	0.991	1.020<br>1.367
Par.	–0.697	0.852 *	0.999	0.641<br>1.132	–0.696	0.852 *	0.999	0.606<br>1.097	–0.700	0.852 *	0.999	0.820<br>0.884	–	–	–	–<br>–

Ins. – Insects. all; Col. – Coleoptera; Hym. – Hymenoptera; Par. – Paraneoptera.

The following parameters was used to run MCMCglmm and PGLS: lambda – 1; prior – V=1, nu=0.002; number of iterations – 240 000; length of burnin – 40 000; amount of thinning – 100; convergence of the chains were analyzed using the Gelman and Rubin Multiple Sequence Diagnostic, Grafen transformation make the phylogeny ultrametric.

* p–value for slope different from 1. 0.05≤p<0.1; ** 0.01≤p<0.05; *** p<0.01

Table S3. Scaling of relative volume of organs and body volume in adult insects.

Taxon	MA				OLS				PGLS				MCMCglmm			
	Elevation	Slope	R^2	CI for slope 95%	Elevation	Slope	R^2	CI for slope 95%	Intercept	Slope	R^2	CI for slope 95%	Elevation	Slope	R^2	CI for slope 95%
**Skeleton**																
Ins.	–0.903	–0.032	0.073	–0.085<br>0.021	–0.904	–0.031	0.073	–0.084<br>0.020	–1.009	–0.012	0.009	–0.072<br>0.048	–0.959	–0.031	0.045	–0.081<br>0.015
Col.	–0.833	–0.034	0.266	–0.071<br>0.001	–0.833	–0.034 *	0.266	–0.070<br>0.001	–0.889	–0.011	0.007	–0.089<br>0.067	–0.835	–0.035	0.206	–0.083<br>0.011
Hym.	–1.105	0.051 *	0.195	–0.296<br>0.411	–1.105	0.050	0.195	–0.261<br>0.362	–1.051	0.044	0.965	0.028<br>0.060	–	–	–	–<br>–
Par.	–0.830	–0.113	0.169	–<br>–	–0.843	–0.106	0.169	–3.109<br>2.896	–0.796	–0.111	0.242	–0.499<br>0.277	–	–	–	–<br>–
**Musculature**																
Ins.	–0.824	0.029	0.040	0.029<br>0.095	–0.823	0.029	0.040	–0.034<br>0.093	–0.929	0.057	0.099	–0.021<br>0.135	–0.911	0.049	0.044	–0.015<br>0.123
Col.	–0.830	0.034	0.072	–0.043<br>0.112	–0.829	0.033	0.072	–0.042<br>0.110	–0.900	0.059	0.104	–0.044<br>0.162	–0.855	0.044	0.084	–0.048<br>0.129
Hym.	–0.679	–0.027	0.153	–0.150<br>0.093	–0.680	–0.027	0.153	–0.147<br>0.091	–0.681	–0.033	0.289	–0.106<br>0.040	–	–	–	–<br>–
Par.	–1.672	0.367 *	0.983	–<br>–	–1.670	0.367 *	0.983	–0.242<br>0.976	–1.661	0.366 *	0.988	0.287<br>0.445	–	–	–	–<br>–
**Digestive system**																
Ins.	–1.289	0.101	0.295	0.028<br>0.175	–1.284	0.099***	0.295	0.027<br>0.170	–1.108	0.059	0.098	–0.024<br>0.142	–1.131	0.064 *	0.268	–0.005<br>0.139
Col.	–1.328	0.102	0.371	0.018<br>0.188	–1.325	0.101 **	0.371	0.018<br>0.183	–1.188	0.065	0.107	–0.046<br>0.176	–1.218	0.078 *	0.324	–0.016<br>0.176
Hym.	–1.28	0.007	0.011	–0.222<br>0.238	–1.284	0.007	0.011	–0.210<br>0.225	–1.264	0.012	0.083	–0.067<br>0.091	–	–	–	–<br>–
Par.	–0.615	–0.077	0.688	–<br>–	–0.615	–0.077	0.688	–0.741<br>0.586	–0.626	–0.076	0.753	–0.162<br>0.010	–	–	–	–<br>–
**Malpighian tubules**																
Ins.	–2.356	0.094 ***	0.141	–0.017<br>0.208	–2.348	0.089 *	0.141	–0.016<br>0.195	–2.215	0.028	0.010	–0.105<br>0.161	–2.260	0.063	0.123	–0.052<br>0.176
Col.	–2.637	0.284 **	0.318	0.168<br>0.479	–2.436	0.160 **	0.318	0.004<br>0.316	–2.163	0.055	0.034	–0.127<br>0.237	–2.300	0.133 **	0.273	–0.019<br>0.301
Hym.	–2.223	–0.167	0.479	–0.769<br>0.036	–2.276	–0.115	0.479	–0.482<br>0.251	–2.294	–0.128	0.693	–0.296<br>0.040	–	–	–	–<br>–
Par.	–1.582	–0.250 ***	0.999	–0.259<br>–0.241	–1.582	–0.250 ***	0.999	–0.259<br>–0.241	–1.582	–0.25	1	–0.251<br>–0.249	–	–	–	–<br>–
**Circulatory system and fat body**																
Ins.	–0.389	0.010	0.022	–0.021<br>0.043	–0.389	0.010	0.022	–0.021<br>0.043	–0.397	0.014	0.019	–0.034<br>0.062	–0.390	0.012	0.018	–0.021<br>0.049
Col.	–0.428	0.027	0.100	–0.024<br>0.078	–0.427	0.026	0.100	–0.023<br>0.077	–0.430	0.024	0.046	–0.045<br>0.093	–0.446	0.026	0.081	–0.033<br>0.095
Hym.	–0.310	–0.044 **	0.839	–0.081<br>–0.008	–0.310	–0.044 **	0.839	–0.081<br>–0.008	–0.304	–0.048 **	0.939	–0.065<br>–0.031	–	–	–	–<br>–
Par.	–0.238	–0.066	0.797	–0.637<br>0.464	–0.238	–0.066	0.797	–0.488<br>0.356	–0.245	–0.065	0.846	–0.119<br>–0.011	–	–	–	–<br>–
**Central nervous system**																
Ins.	–0.993	–0.181 ***	0.624	–0.234<br>–0.130	–0.999	–0.178 ***	0.624	–0.229<br>–0.127	–1.000	–0.186 ***	0.352	–0.303<br>–0.069	–0.975	–0.178 ***	0.636	–0.233<br>–0.120
Col.	–1.057	–0.171 ***	0.701	–0.236<br>–0.107	–1.061	–0.169 ***	0.701	–0.232<br>–0.105	–1.038	–0.179 **	0.320	–0.334<br>–0.024	–1.044	–0.175 ***	0.687	–0.249<br>–0.103
Hym.	–0.688	–0.346 ***	0.911	–0.4465<br>–0.252	–0.692	–0.342 ***	0.911	–0.438<br>–0.247	–0.631	–0.435 **	0.999	–0.456<br>–0.414	–0.725	–0.316 **	0.900	–0.510<br>–0.065
Par.	–1.410	–0.041	0.281	–0.136<br>0.051	–1.411	–0.041	0.281	–0.134<br>0.050	–1.459	–0.025	0.236	–0.115<br>0.065	–1.841	0.157	0.238	–0.843<br>1.083
**Brain**																
Ins.	–1.053	–0.322 ***	0.838	–0.371<br>–0.274	–1.063	–0.316 ***	0.838	–0.364<br>–0.268	–1.176	–0.293 ***	0.833	–0.352<br>–0.234	–1.141	–0.286 ***	0.831	–0.344<br>–0.230
Col.	–1.124	–0.307 ***	0.928	–0.351<br>–0.264	–1.128	–0.305 ***	0.928	–0.348<br>–0.262	–1.158	–0.284 ***	0.865	–0.350<br>–0.217	–1.133	–0.292 ***	0.929	–0.352<br>–0.233
Hym.	–0.853	–0.369 ***	0.815	–0.521<br>–0.230	–0.864	–0.359 ***	0.815	–0.498<br>–0.220	–0.696	–0.519 **	0.975	–0.633<br>–0.405	–0.875	–0.345 **	0.797	–0.576<br>–0.123
Par.	–1.705	–0.073	0.477	–0.183<br>0.033	–1.706	–0.073	0.477	–0.180<br>0.033	–1.818	–0.043	0.629	–0.108<br>0.022	–1.909	–0.018	0.422	–0.576<br>0.394
**Reproductive system**																
Ins.	–0.885	–0.052	0.098	–0.121<br>0.016	–0.887	–0.051	0.098	–0.118<br>0.015	–0.679	–0.121 *	0.203	–0.235<br>–0.007	–0.773	–0.081 *	0.240	–0.175<br>0.018
Col.	–0.770	–0.111 **	0.300	–0.220<br>–0.004	–0.775	–0.108 ***	0.300	–0.211<br>–0.004	–0.624	–0.161*	0.328	–0.312<br>–0.010	–0.676	–0.124 **	0.344	–0.262<br>0.002
Hym.	–1.077	0.210 ***	0.863	0.115<br>0.310	–1.076	0.209 ***	0.863	0.113<br>0.305	–1.149	0.235**	0.963	0.171<br>0.299	–1.093	0.207 **	0.763	0.027<br>0.369
Par.	–0.696	–0.147*	0.983	–0.419<br>0.104	–0.696	–0.147 ***	0.983	–0.393<br>0.097	–0.700	–0.147*	0.988	–0.179<br>–0.115	–	–	–	–<br>–

Ins. – Insects. all; Col. – Coleoptera; Hym. – Hymenoptera; Par. – Paraneoptera.

The following parameters was used to run MCMCglmm and PGLS: lambda – 1; prior – V=1, nu=0.002; number of iterations – 240 000; length of burnin – 40 000; amount of thinning – 100; convergence of the chains were analyzed using the Gelman and Rubin Multiple Sequence Diagnostic, Grafen transformation make the phylogeny ultrametric.

* p–value for slope different from 1. $0.05 \le p < 0.1$; ** $0.01 \le p < 0.05$; *** $p < 0.01$
